# Une électrophorèse des protéines sériques insolite dans un contexte de cholangiocarcinome

**DOI:** 10.11604/pamj.2020.35.117.20616

**Published:** 2020-04-14

**Authors:** Aboubacar Dit Tietie Bissan, Amadou Diawara, Raoul Karfo, Aboubakre Teguete, Oumar Tangara, Alpha Guindo, Fatoumata Maïga, Etienne Algiman

**Affiliations:** 1 Université des Sciences, des Techniques, des Technologies de Bamako (USTTB), Faculté de Pharmacie, Bamako, Mali; 2 Laboratoire d'Analyses Médicale Algi, Quinzambougou, Bamako, Mali; 3 UFR Sciences de la Santé, Université Ouaga Joseph Ki-Zerbo, Ouagadougou, Burkina Faso

**Keywords:** Bis-albuminémie, électrophorèse des protéines sériques, cholestase ictérique, cholangiocarcinome, Bisalbuminemia, electrophoresis of serum proteins, cholestatic jaundice, cholangiocarcinoma

## Abstract

L'électrophorèse des protéines sériques revêt un intérêt diagnostic certain. Nous rapportons un cas associant une électrophorèse insolite à une cholestase ictérique compliquant un cholangiocarcinome. Il s'agit d'un patient âgé de 55 ans hospitalisé pour l'exploration d'un ictère cholestatique. L'électrophorèse capillaire a montré une bis-albuminémie objectivée par l'épaississement du bas du pic de l'albumine, et un pic surnuméraire X. Une seconde électrophorèse sur Hydragel a montré la disparition des deux anomalies précitées qui est la preuve de l'origine acquise de cette bis-albuminémie et de la nature lipidique du pic X. L'étiologie la plus probable dans notre cas est la présence de substances interférentes telles que la bilirubine libre (hyperbilirubinémie), les lipides (hyperlipémie) et les acides biliaires. Un tableau de bis-albuminémie dans un tracé électrophorétique similaire à notre cas, devrait suscitée la recherche de pathologies sous-jacentes.

## Abstract (en)

Serum protein electrophoresis is of certain diagnostic interest. We report a case of unusual electrophoresis associated with cholestatic jaundice complicating a cholangiocarcinoma. The study involved a 55-year old patient hospitalized for the exploration of cholestatic jaundice. Capillary electrophoresis showed bisalbuminemia diagnosed based on the thickening of the bottom of the albumin peak and a supernumerary peak X. Second electrophoresis with Hydragel showed the disappearance of the two above abnormalities which was the proof that bisalbuminemia was acquired and of the lipidic nature of the Pic X. In our case, the most likely cause was the presence of interfering substances such as free bilirubin (hyperbilirubinemia), lipids (hyperlipidaemia) and bile acids. Bisalbuminemia in a patient with similar electrophoretic picture should suggest the search for underlying diseases.

## Introduction

L'électrophorèse des protéines sériques a un intérêt diagnostic certain. C'est est un examen biologique fréquemment prescrit pour la mise en évidence d'anomalies qualitatives et/ou quantitatives des protéines sériques [1,2]. Son indication principale et incontestable est le dépistage, à faible coût, d'une immunoglobulinopathie [2]. L'électrophorèse capillaire, comme l'électrophorèse sur gel d'agarose, permet la séparation de 6 fractions de protéines [2]. Des pics irréguliers ayant une étiologie très diverse sont de plus en plus détectés au niveau des différentes fractions en électrophorèse capillaire, grâce à sa plus grande sensibilité. Les auteurs rapportent un cas rare associant une électrophorèse des protéines sériques insolite à une cholestase ictérique compliquant un cholangiocarcinome.

## Patient et observation

**Figure 1:**
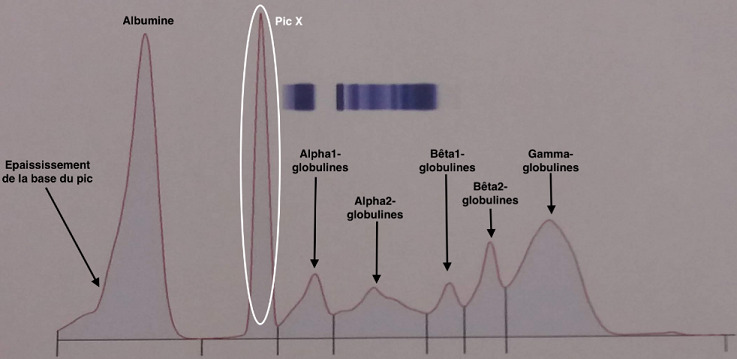
Électrophorèse des protéines sériques objectivant l'épaississement de la base du pic d'albumine et le pic surnuméraire X

**Figure 2:**
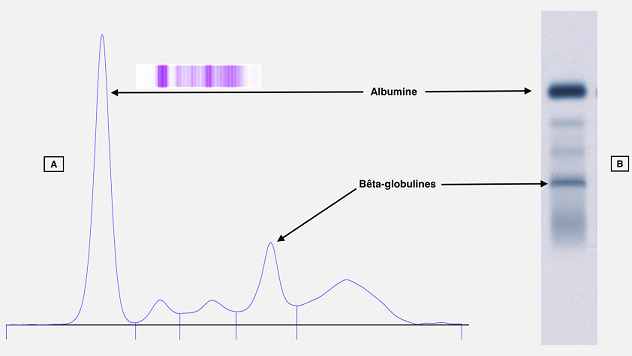
Électrophorèse des protéines sériques sur gel d'agarose (HYDRASYS, SEBIA): A) électrophorégramme obtenu par analyse densitométrique du gel coloré et séché; B) électrophorèse sur gel d'agarose

Nous rapportons le cas d'un patient âgé de 55 ans, connue hypertendue, et diabétique sous insuline. Hospitalisé pour l'exploration d'un ictère cholestatique, la tomodensitométrie thoraco-abdomino-pelvienne a montré un aspect en faveur d'un cholangiocarcinome métastatique avec des métastases pulmonaires, et pancréatiques ainsi qu'une sténose du bas du cholédoque avec une dilatation des voies biliaires. L'examen clinique avait objectivé une asthénie, un ictère cutanéo-muqueux avec un prurit. Le patient présentait depuis une année des douleurs abdominales, une urine décolorée avec une dyspnée d'effort. Le bilan biologique a permis de confirmer la cholestase ictérique avec : phosphatase alcaline (6N), gamma-glutamyl transferase (25N), bilirubine totale (29N), bilirubine direct (37N), bilirubine indirect (16N). Le reste du bilan hépatique a révélé une cytolyse hépatique modérée avec une alanine-amino-transférase (3N), une aspartate-amino-transférase (4N), et une insuffisance hépatocellulaire révélée par une hypoalbuminémie sévère (17,7 g/l) et un taux de prothrombine effondré (29,2%). La lipasémie était normale à 38 UI/l qui atteste de l'absence d'une pancréatite, ainsi que la fonction rénale avec une créatinine normale à 9,8 mg/l (estimation du débit de filtration glomérulaire selon MDRD: 100 ml/mn/1,73 m^2^). On a noté une élévation franche de la Protein Induced by Vitamin K Absence or Antagonist (PIVKA > 30 000Mau/ml), marqueur de grande sensibilité du carcinome hépatocellulaire, mais sans anomalie de l'alpha-foetoprotéinémie. Le bilan lipidique a révélé une hypertriglycéridémie (5,58 g/l), et une hypercholestérolémie totale (23,94 mmol/l) due à une hyperLDLémie (21,30 mmol/l). Le taux d'acide urique était normal avec une glycémie équilibrée (hémoglobine glyquée: 7% sur capillarys 2, Sebia). L'électrophorèse des protéines sériques réalisée par électrophorèse capillaire sur capillarys 2 Sebia, a montré comme anomalies en plus d'une hypoalbuminémie, une bis-albuminémie objectivée par un épaississement du bas du pic de l'albumine sur son versant anodique, un pic atypique surnuméraire X migrant entre les fractions albumine et alpha1-globulines (Figure 1). Une seconde électrophorèse réalisée sur Hydragel Sebia a montré la disparition du pic X ainsi que l'épaississement de la base du pic de l'albumine (Figure 2). Le patient est décédé des suites d'un choc septique.

## Discussion

L'électrophorèse des protéines sériques reste indispensable dans plusieurs démarches diagnostics telles que les gammapathies monoclonales, les processus inflammatoires, les syndromes néphrotiques, etc. Décrite pour la première fois par Scheurlen en 1955 [3], la bis-albuminémie représente une anomalie électrophorétique de l'albumine, caractérisée par un dédoublement de la fraction d'albumine sur le tracé d'électrophorèse des protéines sériques [4,5]. Ces bis-albuminémies ont une prévalence de 0,003% à 0,1% [1,6] avec une incidence qui varie en fonction des populations avec 1/1000 à 1/10 000 chez les caucasiens et les japonais, pouvant atteindre 1% dans certaines tribus amérindiennes [7,8]. Dans notre cas, on note la présence de deux types d'anomalies migrant dans la zone de l'albumine. La première est l'épaississement de la base du pic de l'albumine sur son versant anodique (**Figure 1**) qui correspond à la bis-albuminémie, la deuxième est le pic surnuméraire X dont la migration électrophorétique est plus lente (migration plus cathodique). Par ailleurs, la disparition de ces deux anomalies sur Hydragel (**Figure 2**) est la preuve de l'origine acquise de la bis-albuminémie et du pic X, ainsi que de leur nature différente de l'albumine. En effet, les bis-albuminémies acquises sont transitoires et plus fréquentes que celles d'origine génétiques. On distingue deux causes majeures de bis-albuminémies acquises: une pseudo-bis-albuminémie (aspect artéfactuel) due à la migration de molécules interférentes dans la même zone que l'albumine et une modification de la migration d'une partie de l'albumine dans laquelle l'albumine est de migration plus cathodique (derrière le variant) et présente à une concentration inférieure à la fraction normale [1].

Notre cas est en phase avec la première cause énoncée, compte tenu de la présence de substances interférentes d'origine endogène. En effet, ces substances ont pour cible principale l'albumine en raison de ses propriétés de transport. Une augmentation de la concentration des molécules transportées étant susceptible de modifier l'aspect électrophorétique de l'albumine en général et l'aspect de son pic en particulier [1]. Parmi ces substances endogènes on retrouve: la bilirubine libre, les lipides et les acides biliaires. Produit du catabolisme de l'hème, la bilirubine est transportée par l'albumine au niveau de son site de liaison localisé dans une poche de son sous-domaine IB (faussement appelée bilirubine libre) dans les hépatocytes où elle est conjuguée à l'acide glucuronique pour devenir la bilirubine conjuguée [9] . Dans la réalité, en présence d'une augmentation de « bilirubine libre », le pic d'albumine s'épaissit au niveau de sa base et plus particulièrement sur son versant anodique pour revêtir un aspect de bis-albuminémie acquise. Cet aspect, plus facile à voir en « électrophorèse capillaire » est très rarement décrit dans la littérature [7,9-11]. Nous retrouvons le même aspect de l'albumine (Figure 1) chez notre patient dans un contexte d'augmentation de la bilirubine avec une bilirubine libre à 16N. Les bis-albuminémies dues à des dyslipidémies (hyperlipémie) sont les plus fréquemment rencontrées depuis le développement de l'électrophorèse capillaire, avec laquelle les chilomicrons et les lipoprotéines (Lp) migrent dans la même zone que l'abumine [1]. Cette migration est probablement due à la présence d'un détergent associé au tampon de migration [9]. Notre échantillon a bénéficié d'une seconde électrophorèse sur gel d'agarose où on peut témoigner de la nature lipidique du pic X. En effet, sa migration est plus cathodique par rapport à l'albumine normale en raison de la migration préférentielle des Lp au niveau alpha2 ou bêta1. La Figure 2 montre l'aspect de l'électrophorèse sur gel d'agarose avec disparition du pic X (pouvant maintenant être nommé Lp X) et augmentation de la fraction des bêta-globulines. Ce cas de pseudo-bis-albuminémie acquise au cours d'un ictère cholestatique compliquant un cholangiocarcinome métastatique est l'un des rares cas décrit dans la littérature. En effet, Rar L. *et al*. [12] ont décrit un cas similaire où la différence majeure se situe au niveau de la migration du variant d'albumine qui se situait sur le versant cathodique de la fraction normale de l'albumine et se caractérisait par un épaulement et absence de pic Lp X de migration lente.

## Conclusion

Les pics irréguliers sont de plus en plus fréquents au niveau des différentes fractions en électrophorèse capillaire, spécifiquement les bis-albuminémies. Notre cas clinique affirme encore plus la diversité des interférences pouvant affectées la migration de l'albumine ainsi que la présence de pics divers dans des contextes cliniques bien précis. Les bis-albuminémies acquises et les pics de nature lipidiques au cours des cholangiocarcinomes ne devraient plus être méconnues. Ne présentant pas de caractère pathologique particulier, les bis-albuminémies ainsi que les tracés électrophorétiques atypiques devraient suscitées la recherche d'une pathologie associée.

## Conflits d’intérêts

Les auteurs ne déclarent aucun conflits d'intérêts.
